# Air pollution from traffic and cancer incidence: a Danish cohort study

**DOI:** 10.1186/1476-069X-10-67

**Published:** 2011-07-19

**Authors:** Ole Raaschou-Nielsen, Zorana J Andersen, Martin Hvidberg, Steen S Jensen, Matthias Ketzel, Mette Sørensen, Johnni Hansen, Steffen Loft, Kim Overvad, Anne Tjønneland

**Affiliations:** 1 Institute of Cancer Epidemiology, Danish Cancer Society, Strandboulevarden 49, 2100 Copenhagen, Denmark; 2 Department for Atmospheric Environment, National Environmental Research Institute, Aarhus University, Denmark; 3 Section of Environmental Health, Department of Public Health, University of Copenhagen, Denmark; 4 Department of Epidemiology, Institute of Public Health, Aarhus University, Denmark

## Abstract

**Background:**

Vehicle engine exhaust includes ultrafine particles with a large surface area and containing absorbed polycyclic aromatic hydrocarbons, transition metals and other substances. Ultrafine particles and soluble chemicals can be transported from the airways to other organs, such as the liver, kidneys, and brain. Our aim was to investigate whether air pollution from traffic is associated with risk for other cancers than lung cancer.

**Methods:**

We followed up 54,304 participants in the Danish Diet Cancer and Health cohort for 20 selected cancers in the Danish Cancer Registry, from enrolment in 1993-1997 until 2006, and traced their residential addresses from 1971 onwards in the Central Population Registry. We used modeled concentration of nitrogen oxides (NO_x_) and amount of traffic at the residence as indicators of traffic-related air pollution and used Cox models to estimate incidence rate ratios (IRRs) after adjustment for potential confounders.

**Results:**

NO_x _at the residence was significantly associated with risks for cervical cancer (IRR, 2.45; 95% confidence interval [CI], 1.01;5.93, per 100 μg/m^3 ^NO_x_) and brain cancer (IRR, 2.28; 95% CI, 1.25;4.19, per 100 μg/m^3 ^NO_x_).

**Conclusions:**

This hypothesis-generating study indicates that traffic-related air pollution might increase the risks for cervical and brain cancer, which should be tested in future studies.

## Background

It has been known for decades that urban air is polluted by mutagenic and carcinogenic substances [1], although at concentrations much lower than those in e.g. cigarette smoke and certain work environments. Nielsen et al. [2] found that the concentrations of mutagenic polycyclic aromatic hydrocarbons (PAHs) in Copenhagen were similar to those in other cities in industrialized countries and concluded that traffic was the major source of PAHs in Copenhagen in the early 1990s. Ubiquitous air pollution with low levels of carcinogens is a public health concern, because large populations are exposed; therefore, even a marginally increased risk for cancer at the individual level would result in many cases at the population level.

Ultrafine particles, < 100 nm in diameter, have received much attention since the 1990s because of their high numbers and large surface area [3]. They constitute about 50% of the total surface area of deposited particles in the lung [4]. The airways are the primary target organs, but accumulating evidence from experiments in animals shows that ultrafine particles can translocate to other organs, such as the liver, kidneys, heart and brain [5-7]. Although the number of particles that accumulate in secondary target organs is several orders of magnitude lower than the lung dose, it may not be negligible for carcinogenic processes [4,8].

Previous epidemiological studies have shown associations between ambient air pollution and risk for lung cancer [9-13], but other cancers might also be associated with exposure to polluted air. Cancers of the mouth, pharynx, and larynx are strongly related to smoking and might therefore also be related to other sources of air pollution, as indicated by associations with exposure to combusted indoor fuel [14] and occupational exposure to engine exhaust [15-18].

Bladder cancer has been associated with residence in a polluted city area in a few studies of the general population [19,20] and with occupational exposure to air pollution (traffic, engine exhaust, PAHs) in several (but not all) studies [21-24]. Other cancers have been studied only sparsely in relation to air pollution. Occupational exposure to diesel engine exhaust was associated with risks for cervical [17], ovarian [23], and gastric cancer [25], and several studies indicated associations between occupations associated with exposure to air pollution and risk for kidney cancer [15,16,26]. An ecological association was found between ambient air emissions of volatile organic compounds and brain cancer incidence in Indiana, USA [27], and a recent study indicated that air pollution at the residence increased the risk for breast cancer [28]. Benzene at relatively high occupational concentrations is a known leukemogen, and a few studies have suggested that ambient concentrations near point sources [29] and traffic [30] might be associated with risk for hematological cancers.

We have recently reported on traffic-related air pollution and lung cancer in a large Danish cohort [13]. The individual-level assessment of exposure for all cohort members facilitates a hypothesis-generating screening of possible associations with other cancers than lung cancer. The aim of the study reported here was to investigate whether air pollution from traffic at the residence was associated with risks for 20 selected, relatively frequent cancers in a large Danish cohort.

## Methods

### Design and study participants

During 1993-1997, 57,053 men (48%) and women (52%) aged 50-64 years and living in Copenhagen and Aarhus areas were recruited into the Diet, Cancer and Health cohort study [31]. The baseline examination included a self-administered questionnaire on dietary habits, which covered 192 food and beverage items. The participants also filled in a questionnaire on smoking habits (status, intensity, and duration), occupation, length of school attendance, reproductive factors, history of diseases and medication, and a number of other health-related items [31]. Smoking intensity was calculated by equating a cigarette to 1 g, a cheroot or a pipe to 3 g, and a cigar to 4.5 g of tobacco. Staff in the study clinics obtained anthropometric measurements, including height and weight. Relevant Danish ethical committees and data protection agencies approved the study, and written informed consent was obtained from all participants.

Each cohort member was followed up for cancer occurrence until 27 June 2006 in the Danish Cancer Registry [32] and the Danish Pathology Data Bank by use of the unique personal identification number. We traced the date of death, emigration, or disappearance and retrieved the addresses of each cohort member between 1 January 1971 and 27 June 2006 in the Central Population Registry by use of the personal identification number. The dates of moving into and leaving each address were noted, and the addresses were linked to the Danish address database to obtain geographical coordinates (denoted in the following as 'geocodes'), which were obtained for 94% of the addresses.

### Exposure assessment

The outdoor concentration of NO_x _was calculated for each year at the residential addresses of each cohort member with the Danish AirGIS modeling system (see http://www.dmu.dk/en/air/models/airgis/ and [33]). AirGIS is based on a geographical information system and provides estimates of traffic-related air pollution with high temporal and address level spatial resolution. Air pollution at a location is calculated as the sum of three contributors: (1) local air pollution from street traffic, calculated from input data on traffic (intensity and type), emission factors for the car fleet, street and building geometry, and meteorology; (2) urban background, calculated from data on urban vehicle emission density, city dimensions, and building heights; and (3) regional background, estimated from trends at rural monitoring stations and from national vehicle emissions.

Input data for the AirGIS system were established from various sources and were integrated into the model. A geographical information system (GIS) road network, including construction year and traffic data for the period 1960-2005, was developed and a database on emission factors for the Danish car fleet, with data on light- and heavy-duty vehicles back to 1960, was built and entered into the emission module of the street pollution model. The national topographic GIS database of buildings was supplemented by the construction year and building height from the national Building and Dwelling Register, which provided the correct street and building geometry for a given year at a given address. The geocodes of an address refer to the location of the front door with a precision within 5 m for most addresses. With the geocode of an address and a specified year as the starting point, the AirGIS system automatically generates street configuration data for the street pollution model, including street orientation, street width, building heights in wind sectors, traffic amount, speed and type as well as other data required as inputs for the modeling system. Air pollution is calculated in 2 m height at the façade of the address building. The AirGIS system has been successfully validated in several studies [34-36] and the correlation between modeled and measured 1/2-year mean NO_2 _concentrations at 204 positions in the greater Copenhagen area showed a correlation coefficient (r) of 0.90 with measured concentrations being on average 11% lower than the modeled [35]. We also compared modeled and measured one-month mean concentrations of NO_x _and NO_2 _over a 12-year period (1995-2006) in a busy street in Copenhagen (Jagtvej, 25,000 vehicles per day, street canyon), which showed correlation coefficients (r) of 0.88 for NO_x _and 0.67 for NO_2_. The modeled mean concentration over the whole 12-year period was 6% lower than the measured concentrations for NO_x _and 12% lower for NO_2 _[36]. Thus, the model predicted both geographical and temporal variation well.

We used the concentration of nitrogen oxides (NO_x_) as an indicator of air pollution from traffic because NO_x _level correlates strongly with other traffic-related pollutants in Danish streets, such as particles: *r *= 0.93 for total particle number concentration (size, 10-700 nm) and *r *= 0.70 for particles with a diameter < 10 μm [37]. We calculated the time-weighted average NO_x _concentration at all addresses from 1 January 1971 until cancer diagnosis, censoring, or end of follow-up and entered it as a time-dependent variable into the statistical cancer risk model. If NO_x _could not be calculated because of failed geocoding of an address, we imputed the concentration from that calculated at the preceding address, or that at the subsequent address if the NO_x _concentration was missing for the first address. We included only participants for whom the residential addresses were known and geocoded for 80% or more of the time between 1 January 1971 and censoring, i.e. persons for whom NO_x _concentrations were imputed for less than 20% of the time.

We used the geocode of the address at the time of enrolment into the cohort and the GIS road network with traffic data to derive two variables indicating the amount of traffic near the residence: presence of a street with a traffic density > 10,000 vehicles per day within 50 m of the residence, and the total number of kilometers driven by vehicles within 200 m of the residence each day.

We considered the calculated NO_x _concentration as our primary exposure variable because it takes into account a number of factors that affect traffic-related air pollution and because it reflects exposure over several decades. The two supplementary measures of traffic at the residence are simple indicators that reflect only the time of enrolment into the cohort. The three exposure indicators correlated moderately, with correlation coefficients of 0.53 between calculated NO_x _and presence of a major road within 50 m, 0.43 between calculated NO_x _and traffic load within 200 m, and 0.43 between presence of a major road within 50 m and traffic load within 200 m. We gave most weight to the NO_x _measure in interpreting the results, so that the results for the two traffic indicators could strengthen or weaken interpretation of an effect of NO_x _as a traffic-related air polluter.

The Danish AirGIS modeling system cannot provide reliable estimates for historical particulate matter concentrations because the required input data on historical urban background concentrations and historical emission factors for the Danish car fleet are not available.

### Statistical methods

The end-points for the risk analyses were first primary cancers others than lung cancer. We included only cancer types of which there were more than 30 cases during follow-up. Incidence rate ratios (IRRs) were estimated with Cox proportional hazards models, and 95% confidence intervals (CIs) were calculated on the basis of the Wald test. Age was the time scale, which ensured that the risk estimates were based on comparisons of individuals at exactly the same age, and analyses were corrected for delayed entry at the time of enrolment. People with a cancer diagnosis before entry were excluded from the analyses. Participants were censored at the time of death, the time of loss to follow-up due to emigration or disappearance, the time of a cancer diagnosis other than that under study, or 27 June 2006 (end of follow-up), whichever came first.

The analyses were adjusted for potential confounding factors defined a priori for each cancer site on the basis of two criteria: 1) being an established or likely risk factor for the cancer and 2) data being available. These were: smoking status (never, former, current), smoking intensity (lifetime average, linear), smoking duration (linear), environmental tobacco smoke (dichotomous, no or low, i.e. "no smoker in the home and environmental tobacco smoke at work for less than 4 h/day", versus high), length of school attendance (< 8, 8-10 and > 10 years), physical activity during leisure time (sports: yes/no and h/week for active people (linear)), body mass index (kg/m^2^; linear), dietary intake of fruit (linear), vegetables (linear), red meat (linear), fiber (linear), selenium (sum of diet and supplements; linear), calcium (sum of diet and supplements; linear), alcohol intake (yes/no and g/day (linear)), use of hormone replacement therapy (never/ever and duration for ever users (linear)), use of oral contraceptives (never/ever and duration for ever users (linear)), number of childbirths (none/any and number (linear)), age at first childbirth (none/any and age (linear)), lactation (none/any and time (linear)), previous benign breast tumor (yes/no), previous diagnosis of hypertension (yes/no), skin reaction to sun (severe or moderate burning, light to no burn), tanning during summer (very or moderately dark, faint or not tanned), nevi (no or few, moderate or many) and freckles (none or few, some or many). Moreover, we defined dichotomous indicators of exposure to occupational carcinogens specific to each cancer site from questionnaire responses about jobs held for a minimum of 1 year and from evaluations in the International Agency for Research on Cancer series http://monographs.iarc.fr/ (see Additional file 1).

We tested the linearity of the adjusted associations between NO_x _concentration and risk for each of the 20 cancers by the likelihood ratio test, i.e. testing whether adding non-linear terms improved the fit over the linear model; p < 0.05 was used as criterion for non-linearity. The exposure-response function for 19 sites did not deviate significantly from linearity, while a deviation of borderline significance was found for pancreas cancer. Thus, for all 20 cancers we estimated the IRRs as linear functions per 100-μg/m^3 ^increment in NO_x _and per 10^4 ^vehicle km/day traffic load within 200 m of the residence. Non-linear exposure-response curves with 95% confidence limits are presented graphically for selected cancers. These functions were estimated with the cph function, survival library, R statistical software 2.9.0 using restricted cubic spline in the coxph function. The plots were produced with the plot function in the design library and reflect exposure-response functions after adjustment for cancer-specific sets of potential confounders.

## Results

Of the 57,053 cohort members, 571 were excluded because of a cancer diagnosis before enrolment, 2 because of uncertain date of cancer diagnosis, 960 for which an address history was not available in the Central Population Registry or their baseline address could not be geocoded, and 1,216 because exposure was assessed for less than 80% of the time between 1 January 1971 and diagnosis or censoring. Table 1 shows the baseline characteristics of the 54,304 cohort members who were included, who were followed up for an average of 9.6 years. The participants were on average 56.7 years old at enrolment, and there were slightly more women than men. About one third had never smoked; the median duration of smoking among ever smokers was 33 years. The median time-weighted average NO_x _concentration at the residences between 1971 and the censoring date was 21.9 μg/m^3 ^(minimum, 13.8 μg/m^3^; maximum, 347 μg/m^3^). At enrolment, 8.3% of the cohort members lived at a residence within 50 m of a street with a traffic density > 10,000 vehicles per day.

**Table 1:** Characteristics of 54,304 study participants at baseline and NO_x _concentrations and traffic at their residences

Characteristic	No. (%)	Mean/median (5th-95th percentile)
Age at enrolment (years)		56.7/56.2 (50.7-64.2)

Gender		

Male	25869 (47.6%)	

Female	28435 (52.4%)	

Length of education (years)		

< 8	17996 (33.1%)	

8-10	24994 (46.0%)	

> 10	11255 (20.7%)	

Sport activity in leisure time		

No	25149 (46.3%)	

Yes	29123 (53.6%)	

Hours/week among active		2.4/2.0 (0.5-7.0)

Body mass index		26.1/25.5 (20.4-33.4)

Fruit intake (g/day)		176.6/140.3 (19.0-467.4)

Vegetable intake (g/day)		173.2/157.8 (47.8-352.7)

Alcohol intake		

Abstainers	1256 (2.3%)	

Drink alcohol	53048 (97.7%)	

Amount of alcohol (g/day)^a^		20.0/13.3 (1.1-65.0)

Hormone replacement therapy^b^		

Never	11835 (41.6%)	

Ever	16328 (57.4%)	

Duration of use (years)^c^		7.9/6.0 (2.0-20.0)

Smoking		

Never	19081 (35.1%)	

Former	15600 (28.7%)	

Current	19557 (36.0%)	

Intensity (g/day)^d^		16.3/14.8 (3.8-34.4)

Duration (years)^d^		29.5/33.0 (6.0-46.0)

Environmental tobacco smoke		

No/low	19268 (35.5%)	

High	34768 (64.0%)	

NO_x _at front door ^e ^(μg/m^3^)		28.4/21.9 (14.8-69.4)

Major road^f ^within 50 m		

No	49813 (91.7%)	

Yes	4491 (8.3%)	

Traffic load within 200 m (10^3 ^vehicle km/day)		4.7/2.6 (0.28-15.5)

^a ^Among those drinking alcohol

^b ^For 28,163 women for whom there was information on both present and past use

^c ^Among ever users

^d ^Smoking intensity and duration among ever smokers

^e ^Time-weighted average for the period 1 January 1971 to the censoring date

^f ^More than 10,000 vehicles per day

Table 2 shows the IRRs of 20 cancers in association with concentrations of NO_x _at the residence. Table 3 shows IRRs in association with amount of traffic at the residence. In the adjusted analyses, three sites showed significant associations: primary liver cancer in association with traffic within 200 m of the residence, cervical cancer in association with NO_x _at the residence, a major street within 50 m of the residence and traffic within 200 m of the residence, and brain cancer in association with NO_x _at the residence and a major street within 50 m of the residence.

**Table 2:** Incidence rate ratios for cancer in association with NO_x _at the residence from 1971 onwards

Cancer site (ICD-7)	IR^a^	N^b^	N_cases_^c^	**Incidence rate ratio (95% CI)**, per 100 μg/m^3 ^NO_x_		Adjustment variables^d^
				
				Crude	Adjusted	
Buccal cavity and pharynx (140-148)	0.19	53177	94	1.94 (1.01;3.76)	1.63 (0.79;3.37)	Smoking^e^, education, fruit, alcohol, occupation

Esophagus (150)	0.15	53177	77	1.62 (0.72;3.62)	1.21 (0.49;2.98)	Smoking, education, fruit, alcohol, occupation

Stomach (151)	0.15	53177	80	0.80 (0.27;2.35)	0.65 (0.21;2.02)	Smoking, education, fruit, vegetables, occupation

Colon (153)	0.81	52609	414	1.11 (0.74;1.67)	0.93 (0.60;1.46)	Smoking, physical activity, red meat, fiber, alcohol, BMI, HRT, occupation

Rectum (154)	0.47	52609	246	0.83 (0.46;1.50)	0.80 (0.43;1.48)	Smoking, physical activity, red meat, fiber, alcohol, BMI, HRT, occupation

Liver (155.0)	0.10	54160	57	2.14 (0.96;4.75)	1.66 (0.70;3.94)	Smoking status, alcohol, education, occupation

Pancreas (157)	0.21	54171	112	0.70 (0.27;1.83)	0.64 (0.24;1.71)	Smoking status, BMI, education, occupation

Larynx (161)	0.11	53177	64	1.22 (0.45;3.31)	0.80 (0.26;2.46)	Smoking, education, fruit, alcohol, occupation

Breast (170)	3.57	27735	987	1.39 (1.09;1.77)	1.16 (0.89;1.51)	BMI, education, alcohol, childbirths (number and age at first), lactation, HRT, benign breast disease, physical activity, occupation

Cervix (171)	0.13	27678	35	2.78 (1.18;6.58)	2.45 (1.01;5.93)	Smoking, education, oral contraceptives

Uteri (172)	0.62	27836	171	1.30 (0.71;2.35)	1.15 (0.60;2.21)	HRT, oral contraceptives, BMI, physical activity, number of childbirths, smoking status

Ovary (175)	0.40	28157	111	0.88 (0.36;2.13)	0.81 (0.33;1.99)	Number of childbirths, oral contraceptives, HRT, lactation, occupation

Prostate (177)	2.61	25803	673	0.97 (0.68;1.38)	0.96 (0.67;1.37)	Education, selenium intake, calcium intake, occupation

Kidney (180)	0.20	46259	95	2.14 (1.21;3.79)	1.73 (0.89;3.73)	BMI, smoking, hypertension, education, occupation

Bladder (181)	0.42	53234	221	1.54 (0.96;2.46)	1.32 (0.80;2.19)	Smoking, education, occupation

Melanoma (190)	0.42	53964	226	0.50 (0.23;1.07)	0.52 (0.24;1.11)	Education, skin reaction, tanning, nevi, freckles

Brain (193)	0.17	54304	95	2.28 (1.24;4.17)	2.28 (1.25;4.19)	Occupation

Non-Hodgkin lymphoma (200, 202)	0.36	54245	197	1.11 (0.61;2.03)	1.11 (0.61;2.03)	Education, occupation

Myeloma (203)	0.12	54262	68	0.31 (0.06;1.56)	0.31 (0.06;1.56)	BMI

Leukemia (204)	0.21	54238	117	0.44 (0.15;1.33)	0.47 (0.16;1.39)	Smoking status, occupation

BMI, body mass index; HRT, hormone replacement therapy

^a ^Crude incidence rate per 1,000 person-years for the full cohort, i.e. before exclusions because of failed exposure assessment or missing information on potential confounders

^b ^Number of cohort members contributing to the analyses, i.e. without missing information about exposure or any of the potential confounders

^c ^Number of cases contributing to the analyses, i.e. without missing information about exposure or any of the potential confounders

^d ^See Methods section for further specification

^e ^Adjustment for smoking status, intensity and duration (if not otherwise specified)

**Table 3:** Incidence rate ratios for cancer in association with markers of traffic at residence at the time of enrolment into the cohort between 1993 and 1997

Cancer site (ICD-7)	Incidence rate ratio^a ^(95% CI)			
	
	Major street within 50 m (yes versus no)		Per 10^4 ^vehicle km/day within 200 m	
	
	Crude	Adjusted^b^	Crude	Adjusted^b^
Buccal cavity and pharynx (140-148)	0.92 (0.45;1.90)	0.85 (0.41;1.77)	0.98 (0.68;1.41)	0.87 (0.59;1.29)

Esophagus (150)	1.59 (0.82;3.10)	1.38 (0.71;2.68)	1.20 (0.84;1.72)	1.07 (0.73;1.58)

Stomach (151)	1.01 (0.46;2.19)	0.92 (0.42;1.98)	1.08 (0.74;1.58)	1.00 (0.70;1.48)

Colon (153)	1.13 (0.82;1.55)	0.89 (0.41;1.95)	1.04 (0.88;1.23)	0.99 (0.66;1.47)

Rectum (154)	1.03 (0.67;1.58)	1.00 (0.64;1.56)	0.94 (0.75;1.18)	0.92 (0.72;1.16)

Liver (155.0)	1.58 (0.74;3.34)	1.40 (0.66;2.98)	1.55 (1.09;2.20)	1.45 (1.00;2.09)

Pancreas (157)	0.92 (0.47;1.82)	0.79 (0.38;1.63)	0.78 (0.53;1.14)	0.73 (0.49;1.09)

Larynx (161)	1.24 (0.56;2.72)	1.03 (0.47;2.27)	1.28 (0.88;1.87)	1.13 (0.75;1.70)

Breast (170)	1.11 (0.90;1.38)	0.98 (0.78;1.22)	1.08 (0.98;1.21)	0.98 (0.88;1.10)

Cervix (171)	4.67 (2.29;9.52)	4.36 (2.12;8.95)	1.88 (1.27;2.79)	1.70 (1.12;2.58)

Uteri (172)	1.15 (0.70;1.90)	0.96 (0.55;1.66)	1.19 (0.94;1.52)	1.15 (0.90;1.49)

Ovary (175)	0.50 (0.20;1.23)	0.49 (0.20;1.19)	0.88 (0.61;1.26)	0.80 (0.54;1.17)

Prostate (177)	0.88 (0.67;1.17)	0.91 (0.69;1.21)	0.91 (0.79;1.05)	0.96 (0.83;1.11)

Kidney (180)	1.29 (0.71;2.35)	0.90 (0.44;1.87)	1.10 (0.80;1.51)	1.10 (0.78;1.54)

Bladder (181)	1.06 (0.68;1.66)	0.94 (0.60;1.48)	1.20 (0.97;1.47)	1.09 (0.87;1.35)

Melanoma (190)	0.69 (0.40;1.19)	0.65 (0.37;1.14)	0.83 (0.64;1.08)	0.83 (0.64;1.09)

Brain (193)	1.89 (1.07;3.34)	1.89 (1.07;3.36)	1.27 (0.93;1.75)	1.27 (0.93;1.75)

Non-Hodgkin lymphoma (200, 202)	0.91 (0.54;1.51)	0.90 (0.54;1.51)	1.06 (0.83;1.35)	1.06 (0.83;1.35)

Myeloma (203)	1.06 (0.46;2.45)	1.06 (0.46;2.45)	0.78 (0.48;1.29)	0.78 (0.48;1.29)

Leukemia (204)	0.79 (0.39;1.62)	0.81 (0.39;1.66)	0.73 (0.50;1.09)	0.75 (0.51;1.11)

^a ^Based on same data as the analyses shown in Table 2

^b ^Adjustments identical to those in Table 2

Adjustment for potential confounders decreased the IRRs for many cancers, including some smoking-related cancers, such as esophagus and bladder cancer, and breast cancer, whereas the IRR for e.g. cervical cancer was less strongly affected by adjustment.

**Figure 1:**
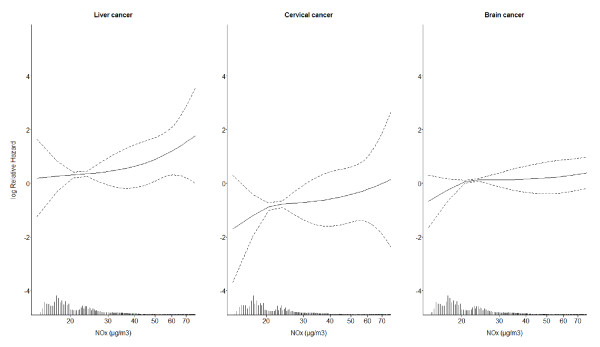
**Non-linear exposure-response functions (filled lines; 95% confidence limits indicated by dashed lines) between average NO_x _concentration (μg/m^3^) at residences from 1971 onwards and risks for primary liver cancer, cervical cancer and brain cancer**. The functions were adjusted for cancer-specific sets of potential confounders, listed in the last column of Table 2. The figure includes the exposure range between the 5^th ^and 95^th ^percentiles (14.8-69.4 μg/m^3 ^NO_x_). The exposure distribution is marked on the x-axis.

Figure 1 shows adjusted exposure-response functions between NO_x _concentration at the residence and risks for each of the three cancers for which significant IRRs are shown in Tables 2, 3. The risk for cervical cancer increased steadily with increasing exposure, the risk for brain cancer increased mostly at concentrations in the lower end of the exposure range, and the risk for liver cancer increased mostly in the upper end of the exposure range.

## Discussion

We found significant associations and exposure-response patterns between traffic-related air pollution at the residence and risks for cervical and brain cancer.

The strengths of this study include a 10-year prospective follow-up of a relatively large cohort and adjustment for potential confounders. Individual assessment of the exposure of all cohort members allowed us to link air pollution to all major types of cancer. Virtually complete follow-up for incident cancers was possible through nationwide population-based registries, and complete follow-up for vital status was available from the Central Population Registry. Another strength of the study is the availability of residential address histories dating back to 1971, so that exposure could be assessed over several decades. A limitation of this study is the relatively few cases of some types of cancer, although more than 100 cases were identified for 11 of the 20 cancers included. The inclusion of cancers at 20 different sites means that the results should be interpreted with caution. The positive findings for cancers at sites for which there is no or little previous epidemiological evidence of an association with air pollution should be considered as the basis for hypothesis-generating.

Exposure assessment is a major challenge in studies of the health effects of long-term exposure to air pollution. We used three markers of air pollution from traffic at residences, which were moderately correlated (*r*, 0.43-0.53). The outdoor NO_x _level at all addresses was calculated over decades from a validated model that requires comprehensive input data; the two other markers are simple, intuitively understandable measures of traffic at the residence at the time of enrolment. The dispersion models we used to assess NO_x _levels at the addresses of study participants have been successfully validated [34-36] and applied [12,13,38]. Although markers of air pollution concentrations are inevitably somewhat uncertain, the resulting non-differential misclassification would create artificial associations only in rare situations [39]. If the geocoding, and therefore also the exposure assessment, failed at an address, we imputed the air pollution concentration from the previous or next address. Since the imputation strategy was identical for all cohort members and the ability of geocoding an address is unlikely to be associated with later development of cancer, we would expect the resulting misclassification of exposure to be non-differential. We minimized the degree of misclassification by including only cohort members for whom air pollution was successfully assessed for at least 80% of the time from 1971 until diagnosis/censoring/end-of-follow up.

This study shows an exposure-response association between concentration of NO_x _at residence and risk for cervical cancer, and associations were also seen for indicators of traffic at the residence. Occupational exposure to diesel engine exhaust was previously associated with risk for cervical cancer in a study with no adjustment for tobacco smoking [17], but to our knowledge no study has been conducted of the exposure of the general population to ambient air pollution. We adjusted our analyses for smoking, education, and oral contraceptive use but had no information on human papillomavirus (HPV) infection, which is a major cause of cervical cancer [40]. It is possible that HPV infection is more prevalent among women living in areas with heavy traffic and air pollution. Early findings of associations between smoking and cervical cancer were similarly suspected of confounding by HPV infection, although today smoking is an established risk factor for this cancer. Further, we cannot exclude the possibility that compliance with the nation-wide cervical cancer screening program differs in areas with high and low levels of air pollution due to differences in educational level. However, the educational level differed only little between cohort members living at addresses with high and low air pollution levels [13] and the results in the present study was adjusted for educational level minimizing the potential for confounding. The hypothesis of an association between air pollution and risk for cervical cancer should be further investigated in a study with control for HPV infection (in addition to other risk factors), preferably with more power than the current study.

We found an exposure-response association between NO_x _at the residence and risk for brain cancer, which was almost doubled for people living close to a street with high traffic density. In general, the causes of brain cancer remain unknown, although high-dose ionizing radiation and certain genetic syndromes are established risk factors [41]. These, however, seem unlikely to be associated with air pollution at the residential address. A previous study in Denmark indicated a higher risk for brain cancer in association with agricultural class and higher income [42]. These factors are probably inversely associated with air pollution from traffic in Denmark, and, if they were risk factors for brain tumors, we would expect any confounding to have decreased the IRR for brain cancer in association with air pollution. There is growing experimental evidence that ultrafine particles can reach the brain both via the systemic circulation through the blood-brain barrier and via the olfactory neuronal pathway [3,5], causing an inflammatory response [43,44]. Further, a recent study showed that exposure to diesel engine exhaust causes functional changes in the human brain indicating cortical stress response [45]. Boeglin et al. [27] showed an ecological association between emissions of volatile organic compounds and brain cancer incidence rates at county level in the USA; but a large cohort study with individual adjustment for potential confounders showed that people who lived in metropolitan areas with higher air pollution levels measured at routine monitoring network stations did not have a higher risk for death from brain cancer [46]. Although our study is smaller, it has several advantages, including individual exposure assessment, thus accounting for within-city variations in air pollution concentrations, which might explain the difference in results. Furthermore, we studied brain cancer incidence, whereas the US study measured mortality. If survival after brain cancer differs in different metropolitan areas and survival correlates with air pollution levels, the results of a mortality study would differ from those of a study of incidence. We recommend that studies be conducted to replicate our finding of an increased incidence of brain cancer in association with individual-level exposure to air pollution.

We found a borderline significantly increased risk for liver cancer associated with traffic within 200 m of the residence, after adjustment for relevant confounders, although there was no significant association with NO_x _levels. There is consistent evidence that liver cancer is associated with tobacco smoking [47]. One of the few previous epidemiological studies on ambient air pollution and liver cancer was a retrospective cohort study, which showed an increased risk in urban bus drivers and tramway employees [16]. Mucociliary clearance of particles deposited in the airways usually leads to gastrointestinal exposure due to swallowing, and, in experimental studies, intragastric exposure of animals to diesel exhaust particles induced oxidative stress and DNA damage in the liver [48]. In addition, particles translocated to the circulation accumulated in Kupfer cells in the liver, with very slow elimination and further potential oxidative stress [49].

Our study also showed that the risk for kidney cancer increased with NO_x _concentration at the residence. Several studies of occupational groups, such as transport workers, drivers, policemen, metal foundry workers, and gasoline service station workers exposed to gasoline vapors, engine exhaust, PAHs, and other air pollutants, have indicated weakly increased risks for kidney cancer [15,16,26], although the literature is neither consistent [23] nor conclusive [50]. The indication in the present study of an association between ambient air pollution at the residence and risk for kidney cancer in a general population should be confirmed before conclusions can be drawn.

Our study showed a weak, insignificant association between traffic-related air pollution and risk for breast cancer. A recent study in Montreal, Canada, showed that the risk for breast cancer was associated with NO_2 _concentrations at the residence [28], and a study in New York, USA, indicated an association between early-life exposure to air pollution at the residence and risk for this cancer [51]. PAH-induced breast tumor mutations might explain any link between air pollution and risk for breast cancer [52].

Previous studies have shown associations between risks for upper aerodigestive tract cancers and indoor fuel combustion [14] and occupational exposure to engine exhaust [15-18], and our study also indicated a possible association between ambient traffic-related air pollution and cancers of the buccal cavity and pharynx, although the result was insignificant.

Our results showed a weak, insignificant association between traffic-related air pollution and bladder cancer. The evidence of an association between ambient air pollution and bladder cancer in the general population is not conclusive [19,20,30].

Benzene at relatively high occupational concentrations is a known leukemogen, and a few studies have suggested that ambient concentrations near point sources [29] and near traffic [30] might be associated with risks for hematological cancers, whereas other studies found no such association [53,54]. The exposure of the general population to benzene is much lower than the lowest effect level seen in studies of occupational exposure, so that any related risk for leukemia in the general population would probably not be detectable with current methods [55]. Our results are in accordance with this notion.

Although we found associations between NO_x _concentration and the risks for some cancers, NO_x _is an indicator of vehicle engine exhaust, which is a complex mixture of many carcinogenic and mutagenic chemicals [1]. The NO_x _concentration correlates closely with that of particulate matter, especially the ultrafine fraction emitted from diesel engines in Danish streets [37]. Although it is difficult to disentangle the effects of single air pollutants in epidemiological designs, particulate matter from traffic emissions appears to be the most important determinant of cancer risk. Ultrafine particles have a large surface area and contain absorbed PAHs, transition metals and other substances, which cause oxidative stress, inflammation and direct and indirect genotoxicity [56,57]. Further, there is evidence that ultrafine particles can translocate from the airways to other organs [7], which might explain our finding of higher risks for cervical and brain cancer in cohort members living at residences with high levels of traffic-related air pollution.

## Conclusions

In conclusion, this cohort study shows significant associations between traffic-related air pollution at residential addresses over several decades and risks for cervical and brain cancer. Although experimental evidence shows that ultrafine particles can translocate from the airways to other organs, our results are based on hypothesis-generating screening of 20 cancers and future epidemiological studies are needed to provide further information on possible risks for cancer associated with traffic-related air pollution. In particular the hypotheses of associations with brain and cervical cancer require further testing.

## List of abbreviations

IRR: incidence rate ratio; CI: confidence interval; PAH: polycyclic aromatic hydrocarbon; GIS: geographical information system.

## Competing interests

The authors declare that they have no competing interests.

## Authors' contributions

ORN conceived and designed the study, participated in acquisition of environmental data and exposure assessment, participated in planning of data analyses and drafted the manuscript. ZA participated in planning of the statistical analyses and performed record linkages, data processing and statistical analyses. MH, SSJ and MK developed the air pollution modeling system and conducted the air pollution calculations. JH defined the occupations associated with risk for each cancer. SL contributed to the manuscript. AT and KO established the Diet Cancer and Health cohort and provided cohort data. All authors participated in interpretation of data, commented on the manuscript and approved the final manuscript.

## Supplementary Material

Additional file 1 **Occupations and jobs associated with risks for each cancer**. Click here for file
