# Supplementary material for: Air pollution from traffic and cancer incidence: a Danish cohort study
Source: Environ Health. 2011 Jul 19;10:67. doi: 10.1186/1476-069X-10-67 (PMC3157417; doi:10.1186/1476-069X-10-67)
Supplement: Additional file 1 — Occupations and jobs associated with risks for each cancer. [file 1476-069X-10-67-S1.DOC]

**Additional file 1**

**Occupations and jobs associated with risks for each cancer**

*Buccal cavity*: Shipyard, glass industry, manufacture of asbestos or asbestos cement, asbestos insulation, waiter or cook

*Esophagus*: Rubber industry, waiter or cook

*Stomach*: Shipyard, glass industry, manufacture of asbestos or asbestos cement, asbestos insulation

*Colon*: Shipyard, glass industry, manufacture of asbestos or asbestos cement, asbestos insulation, waiter or cook

*Rectum*: Shipyard, glass industry, manufacture of asbestos or asbestos cement, asbestos insulation, waiter or cook

*Liver*: Waiter or cook

*Pancreas*: Waiter or cook

*Larynx*: Rubber industry, shipyard, glass industry, manufacture of asbestos or asbestos cement, asbestos insulation

*Lung*: Mining, rubber industry, leather tannery, manufacture of shoes or leather products, metal processing (welding, painting, electroplating), foundry, steel-rolling mill, shipyard, glass industry, building industry (roof constructor, asphalt worker, demolition worker), truck, bus or taxi driver, manufacture of asbestos or asbestos cement, asbestos insulation, cement article industry, china and pottery industry, butcher, painter, welder, auto mechanic, waiter or cook

*Breast*: Health service

*Cervix*: None

*Uteri*: None

*Ovary*: Shipyard, manufacture of asbestos or asbestos cement, asbestos insulation

*Prostate*: Rubber industry

*Kidney*: Waiter or cook

*Bladder*: Chemical industry (dyeworks), rubber industry, textile industry (dyeworks), metal processing (painting, coating), glass industry, truck, bus or taxi driver, painter, hairdresser, waiter or cook

*Melanoma*: None

*Brain*: Chemical industry (oil refinery)

*Non-Hodgkin lymphoma*: Rubber industry

*Myeloma*: None

*Leukemia*: Chemical industry (oil refinery), rubber industry

Table A1. Cohort participants ever occupied for at least 1 year in an industry or job associated with risk (see above) for the specified cancer

|  | Number (%) |
| --- | --- |
| All | 54 304 (100) |
| Buccal cavity | 4 765 (8.8) |
| Esophagus | 2 854 (5.3) |
| Stomach | 2 447 (4.5) |
| Colon | 4 765 (8.8) |
| Rectum | 4 765 (8.8) |
| Liver | 2 475 (4.5) |
| Pancreas | 2 475 (4.5) |
| Larynx | 2 799 (5.1) |
| Lung | 13 717 (25.3) |
| Melanoma of skin | 227 (4.0) |
| Breast | 6 377 (11.7) |
| Ovary | 2 208 (4.1) |
| Prostate | 419 (0.8) |
| Kidney | 2 475 (4.5) |
| Bladder | 8 516 (15.7) |
| Brain | 240 (0.4) |
| Non-Hodgkin lymphoma | 419 (0.8) |
| Leukaemia | 652 (1.2) |
